# Changes in analgesic prescriptions in Dutch general practice

**DOI:** 10.1080/02813432.2024.2387423

**Published:** 2024-08-19

**Authors:** D. Veldkamp, N. Pooters, H. J. Schers, R. Akkermans, T. C. Olde Hartman, A. A. Uijen

**Affiliations:** Department of Primary and Community Care, Radboud University Medical Centre Nijmegen, Nijmegen, The Netherlands

**Keywords:** Analgesics, primary health care, chronic opioid use, risk factors, chronic pain

## Abstract

**Background:**

Increases in opioid prescriptions have been described; however, recent trends and prescribing patterns of analgesics in Dutch general practice are largely unknown.

**Objective:**

To investigate recent changes in the number of analgesic prescriptions, and the indications for prescribing strong opioids. Furthermore, we aim to identify risk factors for chronic opioid use in Dutch general practice.

**Design and setting:**

A retrospective cohort study from 1 July 2013 to 31 June 2022, using a primary care practice based research network.

**Subjects:**

Patients with ≥1 prescription for analgesics during the study period were included.

**Main outcome measure:**

Changes in the number of prescriptions for paracetamol, non-steroidal anti-inflammatory drugs (NSAIDs) and opioids in Dutch general practice during the 9-year study period. Moreover, we analyzed indications for prescribing strong opioids by the general practitioner (GP).

**Results:**

A total of 18,433 analgesic users were identified. Over time, prescriptions for paracetamol, NSAIDs and weak opioids decreased, while the number of strong opioid prescriptions increased. General practitioners prescribed more strong opioids for non-malignant pain, whereas prescriptions for malignant pain remained stable over time. Risk factors for chronic opioid use (≥90 days) included older age, lower educational level, smoking status and having a history of a musculoskeletal or psychological disorder, a malignancy or sexual, physical or psychological abuse.

**Conclusions:**

Considering the increase in strong opioid prescriptions for benign conditions, GPs need to be vigilant for patients who are at risk for chronic use. Regular monitoring and awareness for psychosocial factors in treatment of chronic pain may be key in preventing harms associated with persistent opioid use.

## Introduction

The prevalence of moderate to severe chronic pain among Dutch adults is estimated to be around 18% [1]. Analgesics that are prescribed by the general practitioner (GP) for acute or chronic nociceptive pain are paracetamol, non-steroidal anti-inflammatory drugs (NSAIDs) and opioids [2]. Increases in opioid prescriptions have been described in almost all European countries [3], also in the Netherlands [4,5]. Opioid prescribing rates in the Netherlands are in the middle in relation to other European countries [3]. However, recent numbers from general practice are unknown. Between 2010 and 2017, the number of opioid users has increased by over 55% extramural [6], while research has shown that self-reported pain prevalence and severity remained constant [5]. Previous research indicates an increase in (strong) opioid prescriptions in Dutch primary care, primarily driven by prescriptions for benign pain [7]. Serious adverse events regarding analgesic use are well established [8–11]. So far, risk factors for chronic opioid use in Dutch general practice are unknown. A previous study from 2019 found several risk factors associated with opioid prescription in the Netherlands (e.g. being older than 65 years, having only primary school education, being widowed, feeling symptoms of depression), but did not analyze risk factors for chronic use [12]. It has been estimated that 75% of first prescriptions of opioids originate from the GP [6], which highlights the importance of emerging research regarding these prescriptions in general practice.

This study provides an overview of recent changes in the number of prescriptions for paracetamol, NSAIDs and opioids in Dutch general practice. Indications for prescribing strong opioids by the GP are analyzed. Moreover, we aim to identify risk factors for chronic opioid use.

## Methods

### Setting

We performed a retrospective cohort study using the Family Medicine Network (FaMe-Net) database, a Dutch practice-based research network consisting of eight general practices (40 GPs, nearly 50,000 registered patients) [13]. In FaMe-Net, all morbidity is registered in episodes of care. An episode of care can be defined as ‘a health problem presented by an individual to a healthcare provider, from the first presentation until the last encounter’. The title of an episode of care is the episode diagnosis, classified with the International Classification of Primary Care (ICPC-2). The episode diagnosis can be modified during the episode of care. For example, when abdominal pain turns out to be a colon carcinoma on further diagnosis. Medication prescriptions are recorded using ATC (Anatomical Therapeutic Chemical) codes [14], and GPs are mandated to link the prescription to the relevant episode diagnosis. GPs routinely register the episode diagnosis prior to prescribing the medication. During the 9-year study period, not all patients were continuously included due to various reasons, such as changing to another general practice or death. In addition, not all general practices collected data for the entire study period.

Since 2016, Fame-Net has been systematically gathering personal and contextual characteristics from all enlisted adult patients (>18 years). Patients are invited via email to complete a questionnaire. This questionnaire was developed by literature search and focus groups of patients and GPs. We sent the questionnaire in 2016 to all patients with a known email address, and every half year we sent the questionnaire to newly enlisted patients and patient from who we registered an email address in the last half year. Characteristics that we included were highest level of education, smoking, alcohol use, marital state and having a history of sexual, physical or psychological abuse.

### Study population

We included all enlisted patients ≥18 years of age with one or more prescription for paracetamol, NSAIDs or opioids between 1 July 2013 and 30 June 2022.

### Variables

All included ATC codes by analgesic group are shown in Supplementary Table 1. We included all analgesics in use in the Netherlands, except for codeine (ATC-code N02AA59) and acetylsalicylic acid (ATC-code N02BA51) as these medicines are mainly prescribed for other reasons than pain (i.e. cough and blood thinning respectively). Supplementary Table 2 shows the ICPC-codes for each indication group.

As chronic pain is usually described as pain lasting longer than 3 months [15–19], we measured chronic analgesic use as 90 or more prescription days within a period of 180 days. To indicate chronic opioid users, an algorithm was used that determined for each patient whether this criterion was met. The algorithm took into account the dosage frequency and number of prescribed units (e.g. tablets, capsules, transdermal patches, etc.) of each individual prescription to calculate the duration of each prescription. In case of as-needed prescriptions, maximum usage was assumed. Overlapping or multiple prescriptions were summed up to determine the total number of prescribed units. In case of a missing dosage frequency (5% of prescriptions), this algorithm used the most common dosage based on product type and code.

Based on this algorithm, patients were categorized as chronic or non-chronic opioid users. Each patient could only be included once as an analgesic user in our study.

To identify risk factors for chronic opioid use, we analyzed the following patients’ characteristics: age, sex, lifelong history of diseases, highest achieved educational level, smoking, alcohol consumption, marital state and having a history of sexual, physical or psychological abuse. Age is defined as age at time of the first analgesic prescription during the study period. Age is analyzed as a continuous variable. For history of diseases, we selected diseases with an expected association with chronic opioid use based on prevalence in the dataset, clinical experience and literature [9]. Supplementary Table 3 shows the three groups of diseases with their accompanying ICPC codes. The educational status is categorized into three groups: low, moderate and high levels of education based on the Dutch standard education classification utilized by Statistics Netherlands [20] (Supplementary Table 4). Alcohol consumption is classified as none, moderate or heavy according to definitions of the National Institute on Alcohol Abuse and Alcoholism [21] (Supplementary Table 4).

### Statistical analyses

Descriptive statistics were performed to gain insight into the baseline characteristics of the study population. Mean and standard deviation (SD) or median and interquartile range for continuous characteristics and number and percentages for categorical characteristics were determined. Poisson’s regression analyses were performed to analyze changes over time in the number of analgesic prescriptions and the indications for prescribing strong opioids. The number of prescriptions were expressed per 1000 patient years in the total FaMe-Net database by correcting for the total population size on the first of January in the corresponding year.

A multilevel logistic regression analysis was performed to determine the probability of becoming a chronic opioid user considered the previously mentioned patients’ characteristics. We performed multilevel analysis taking into account the clustering of patients within general practices. A model with a random intercept (the general practice a patient is registered in) and all other variables fixed (patient characteristics) was used.

Patients with missing variables were excluded. Independent-samples *t*-tests were performed to determine the difference in age. A significance level of 5%, based on two-sided testing was used, and all analyses were carried out using SPSS, version 27 (SPSS Inc., Chicago, IL).

## Results

We included 18,433 analgesic users (45.0% of the average population size in the FaMe-Net database during the study period). We found an average of 6.7 prescriptions for analgesics to the included patients during the study period. Table 1 shows the baseline characteristics of the study population. In total, 6.1% of analgesic users were chronic opioid users. Chronic opioid users had a higher age compared to the non-chronic users (*p* < .001). More analgesics were prescribed to women compared to men. The greatest difference is observed in the chronic group: 61.1% of women versus 38.9% of men. In total, 7339 (39.8%) included patients completed the questionnaire about personal and contextual characteristics. Table 2 shows no clinically significant differences in patient characteristics between the total study population and the patients who filled in the context form.

**Table 1.** Baseline characteristics of 18,433 analgesic users.

Characteristics	Analgesic, non-chronic opioid, *n* = 17,308 (93.9%)	Chronic opioid, *n* = 1125 (6.1%)	*p* Value
Age years, mean (SD)	46.8 (19.3)	60.0 (17.3)	<.001
Sex, *n* (%)			.149
Male	7405 (42.8%)	438 (38.9%)	
Female	9905 (57.2%)	687 (61.1%)	
History of diseases, *n* (%)			
Musculoskeletal disorder	4410 (25.5%)	575 (51.1%)	<.001
Psychological disorder	4294 (24.8%)	369 (32.8%)	<.001
Malignancy	1708 (9.9%)	289 (25.7%)	<.001
Contextual factors	Analgesic, non-chronic opioid, *n* = 6922 (94.3%)	Chronic opioid, *n* = 417 (5.7%)	*p* Value
Educational level, *n* (%)			<.001
Low	1424 (20.6%)	144 (34.5%)	
Middle	2289 (33.1%)	144 (34.5%)	
High	3005 (43.4%)	116 (27.8%)	
Unknown	204 (2.9%)	13 (3.1%)	
Smoking, *n* (%)			<.001
Never	3179 (45.9%)	121 (29.0%)	
Former	2593 (37.5%)	184 (44.1%)	
Current	1.089 (15.7%)	106 (25.4%)	
Unknown	61 (0.9%)	6 (1.4%)	
Alcohol consumption, *n* (%)			<.001
None	1628 (23.5%)	163 (39.1%)	
Moderate	4103 (59.3%)	178 (42.7%)	
Heavy	1076 (15.5%)	73 (17.5%)	
Unknown	115 (1.7%)	3 (0.7%)	
Marital state, *n* (%)			.483
Together	5865 (84.7%)	348 (83.5%)	
Single	1057 (15.3%)	69 (16.5%)	
Unknown	0 (0.0%)	0 (0.0%)	
Having a history of sexual, physical or psychological abuse, *n* (%)			<.001
No	5721 (82.6%)	308 (73.9%)	
Yes	1061 (15.3%)	100 (24.0%)	
Unknown	149 (2.0%)	9 (2.2%)	

**Table 2.** Patient characteristics of total study population and of patients who filled in the context form.

	Total included patients	Patients who filled in a context form
Characteristics	*n* = 18,433	*n* = 7399
Age years, mean (SD)	47.6 (19.4)	48.3 (16.0)
Sex, *n* (%)		
Male	7834 (42.5%)	2945 (39.8%)
Female	10,599 (57.5%)	4454 (60.2%)
History of diseases, *n* (%)		
Musculoskeletal disorder	4985 (27.0%)	2013 (27.2%)
Psychological disorder	4663 (25.3%)	2001 (27.0%)
Malignancy	1997 (10.8%)	771 (10.4%)
Chronic opioid use, *n* (%)	1125 (6.1%)	417 (5.7%)

### Number of prescriptions over time

**Figure 1.**
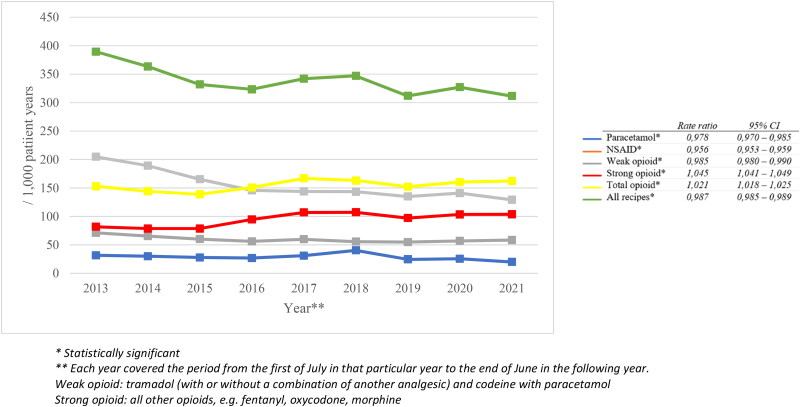
Changes in the number of prescriptions for analgesics per 1000 patient years in the total FaMe-Net database.

**Figure 2.**
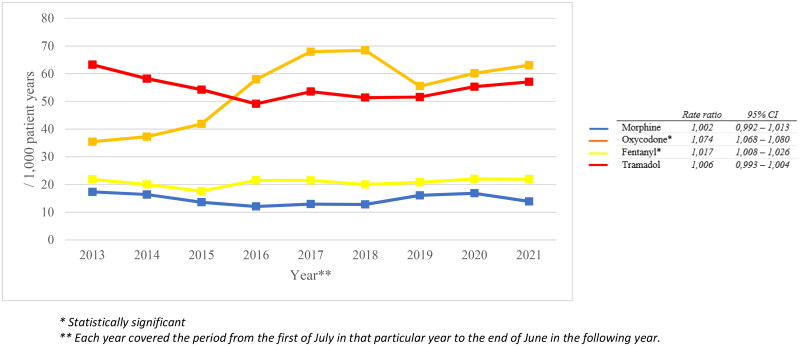
Changes in the number of prescriptions for most commonly prescribed opioids per 1000 patient years in the total FaMe-Net database.

In total, the study population received 124,164 analgesic prescriptions, of which 10,533 for paracetamol (8.5%), 56,703 for NSAIDs (45.7%) and 56,928 for opioids (45.8%). Figure 1 shows that the total number of prescriptions decreased over time (rate ratio 0.987; 95% CI 0.985–0.989), caused by a decrease in prescriptions for paracetamol, NSAIDs and weak opioids. In contrast, the number of strong opioid prescriptions increased over time (rate ratio 1.045; 95% CI 1.041–1.049). In 2014, we observed 152 opioid prescriptions (weak and strong) per 1000 patient years, compared to 159 prescriptions per 1000 patient years in 2021. Figure 2 shows that the number of oxycodone prescriptions increased the most: it nearly doubled between 2013 and 2022.

### Indications for strong opioid prescriptions

**Figure 3.**
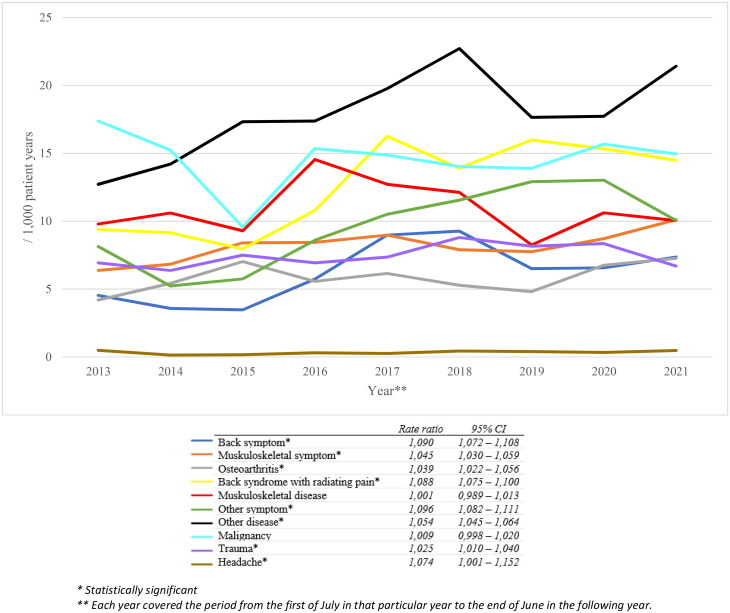
Changes in number of strong opioid recipes within the accompanying groups per 1000 patient years in the total FaMe-Net database.

Over time, strong opioids were more frequently prescribed for all kinds of benign symptoms or diseases, such as back and musculoskeletal symptoms, osteoarthritis, back syndrome with radiating pain, headache and traumas (Figure 3). We found no significant change in the number of strong opioid prescriptions for malignancies over time.

### Risk factors for chronic opioid use

**Figure 4.**
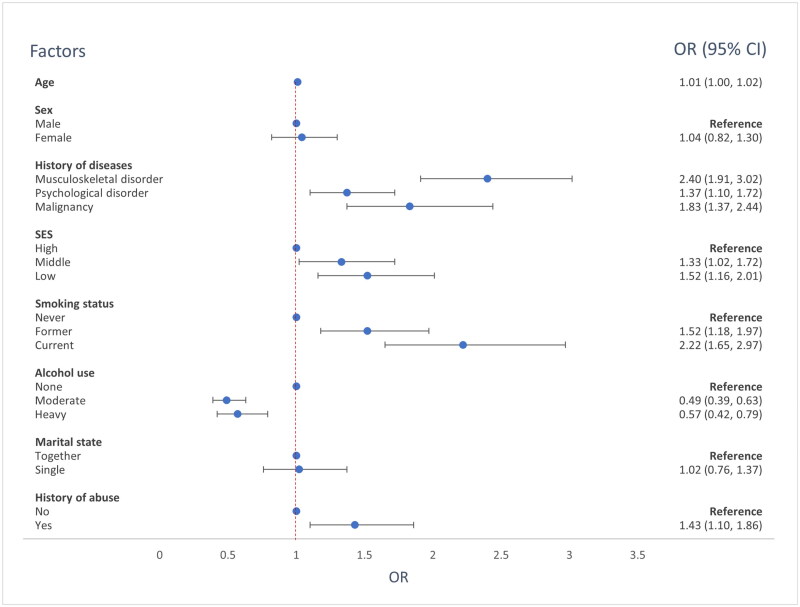
Factors associated with chronic opioid use. OR: odds ratio; 95% CI: 95% confidence interval. An odds ratio <1 shows a negative association between the factor and chronic opioid use, while an odds ratio >1 shows a positive association.

Figure 4 shows that older age, low and middle educational level, smoking and having a history of a musculoskeletal or psychological disorder, a malignancy or sexual, physical or psychological abuse were associated with chronic opioid use. As age is analyzed as a continuous variable, it means that the odds for chronic opioid use increases with 1.01 every year. Moderate and heavy alcohol consumption were negatively associated with chronic opioid use.

Additional results including the model fitting and estimates of fixed and random effects are shown in Supplementary Table 5.

## Discussion

### Summary

This study showed that, between 2013 and 2022, the overall number of prescriptions for paracetamol, NSAIDs and weak opioids decreased, while the number of prescriptions for strong opioids increased. We found that the number of oxycodone prescriptions almost doubled from 2013 to 2022. Strong opioids were more frequently prescribed for benign conditions, whereas the strong opioid prescriptions for malignant pain did not change over time. We identified the following risk factors for chronic opioid use: older age, lower educational level, smoking and having a history of a musculoskeletal or psychological disorder, a malignancy or sexual, physical or psychological abuse.

### Strengths and limitations

The major strength of our study is the large dataset derived from a database where GPs reliably record medication prescriptions linked to relevant diagnoses. This study also has some limitations. First, during the last years of the study, prescriptions from medical specialists were increasingly recorded in the GP electronic patient records. Hence, we probably overestimated the prescriptions of these analgesics by the GP, as we could not identify in the patient record whether the analgesics were prescribed by the medical specialist or the GP. Also, administration errors in prescriptions by GPs may occur. No dosing frequency had been entered in about 5% of prescriptions. In addition, we have assumed maximum use of as-needed prescriptions; however, we do not know the dosage frequency taken by the patient. This may have underestimated the number of chronic opioid users. In our study, we did not account for the dose of prescribed analgesics. However, we do not expect this to have changed over the 9-year study period, as guidelines in prescribing analgesics did not show major changes. This study only included analgesic prescriptions from physicians, while some analgesics are available over the counter (OTC), such as paracetamol and certain NSAIDs. A previous Dutch study estimated an OTC-use of 33% for NSAIDs [22]. Hence, we probably underestimated the use of these analgesics.

Additionally, context factors were obtained from a questionnaire, which was completed by nearly 40% of the study population. Patients with an unknown email address did not receive the questionnaire, potentially introducing response bias as a limitation of our study. Furthermore, data collection began retrospectively from 2013, while the questionnaire was sent out to patients from 2016 onwards. This might result in misclassification bias, as characteristics could have changed between the time of opioid use and questionnaire response. On average, patients completed the questionnaire 2.3 years after enrolment. As this is a relatively short time frame, we expect that most characteristics (e.g. highest level of education, having a history of sexual, physical or psychological abuse) remained stable during this time period.

In total, the mean age of the FaMe-Net population is 37.1 years and 51.2% is female. In comparison, the mean age of the total Dutch population is 42.4 years and 50.3% is female [20]. So, the FaMe-Net population is slightly younger than the general Dutch population. Hence, we might underestimate the change in analgesic prescriptions over the years.

### Comparison with existing literature and interpretation

In line with previous Dutch literature, we observed an increase in strong opioid prescriptions [6,7]. A possible explanation for that increase may be that GPs have become increasingly familiar with the long-term effects of NSAIDs. Moreover, opioids were given a prominent role in the Dutch primary care guideline on pain management in 2016 [6]. However, an opioid crisis like in the United States (US) is fortunately not (yet) the case for the Netherlands. In the US, there were 433 opioid prescriptions per 1000 patient years in 2020 [23], while we observed 158 opioid prescriptions per 1000 patient years in the same year. The increase in opioid prescriptions in the US is suspected to be caused by an increase in the use of oxycodone [24], which is in line with our study where we found a doubling of oxycodone prescriptions. This may be explained by the reintroduction of oxycodone in postoperative pain management guidelines in 2013 [25]. A previous Dutch study found a fourfold increase of oxycodone between 2010 and 2017 extramural [6]. One reason for the smaller increase seen in our study may be due to the difference in time period. In recent years, much attention has been focused on the rise and reduction of opioid prescriptions, which may have made GPs more cautious. In addition, the 2018 revised primary care guideline on pain management advised to be very cautious in prescribing opioids for non-palliative pain [6].

In contrast to the study from Jani et al. [9], we were not able to identify female sex as a risk factor for chronic opioid use. This may be due to the fact that we included female sex in a model with more possible risk factors which could influence one another. In line with previous literature, we found lower educational level as positively associated with chronic opioid use [9,26]. The risk factors older age, musculoskeletal and psychological disorders and smoking are also in accordance with earlier studies [9,10]. It has previously been shown that increasing age, lower levels of education, and smoking are associated with chronic pain [27,28].

Patients having a history of sexual, physical or psychological abuse have higher utilization of health care services [29–31], and chronic pain is more common in that patient group [32]. This possibly contributes to the chronic use of opioids in this patient group. In accordance with our results, previous studies also demonstrate an association between a history of sexual abuse and prescription opioid use [33,34].

Bedene et al. [12] analyzed risk factors for at least one opioid prescription and found among others that higher age, having only primary school education, smoking, reporting feeling symptoms of depression and self-reported musculoskeletal disorders or cancer were associated with a higher risk for opioid prescription. Moreover, they found that an alcohol use disorder was negatively associated with opioid prescription. We analyzed risk factors for chronic opioid use instead of at least one opioid prescription and found comparable risk factor for chronic opioid use. Moreover, we also found that moderate and heavy alcohol consumption were negatively associated with chronic opioid use.

### Implications for practice and research

Further research is needed to determine whether patients experience better pain management due to the stronger analgesics prescribed. On the other hand, recent literature showed an increase in hospitalizations and mortality caused by opioid (ab)use in the Netherlands in recent years [12,24], which is the side note of increased opioid use.

Our study provides individual-level factors for patients who are at risk for chronic use, providing GPs guidance in recognizing patients in whom they should be extra vigilant in opioid treatment. We advise GPs to evaluate opioid therapy more often after the first opioid prescription, especially in these high-risk patients. The Dutch primary care guideline on pain management [2] should pay more attention to extra evaluation moments, especially for high-risk patients, to make Dutch GPs more aware. Subsequently, an evaluation of these changes in the guideline could be a potential future study.

Another potential future study might be to analyze the relation between the duration and intensity of the pain and analgesic prescriptions.

## Conclusions

Between 2013 and 2022, the number of opioid prescriptions for non-malignant pain has increased, while the number of prescriptions for paracetamol, NSAID and weak opioids decreased in Dutch primary care. We identified older age, lower educational level, smoking and having a history of a musculoskeletal or psychological disorder, a malignancy or sexual, physical or psychological abuse as risk factors for chronic opioid use. GPs should be extra cautious when prescribing opioids to these patient groups in order to prevent harms associated with chronic opioid use.

## Supplementary Material

Supplemental Material

## Data Availability

The data underlying this article will be shared on reasonable request from the corresponding author.
