# Supplementary material for: Changes in analgesic prescriptions in Dutch general practice
Source: Scand J Prim Health Care. 2024 Aug 19;42(4):714–22. doi: 10.1080/02813432.2024.2387423 (PMC11552297; doi:10.1080/02813432.2024.2387423)
Supplement: Supplemental Material [file IPRI_A_2387423_SM9656.docx]

**Supplements**

**Table S1:** classification of ATC codes per analgesic group included in the analysis

| **Paracetamol** | **NSAID** | **Weak opioids** | **Strong opioids** |
| --- | --- | --- | --- |
| N02BB02 = metamizole sodium | M01AA01 = phenylbutazone | N02AX52 = tramadol, combinations | N02AX06 = tapentadol |
| N02BE01 = paracetamol | M01AB01 = indomethacin | N02AX02 = tramadol | N02AE01 = buprenorphine |
| N02BE51 = paracetamol, combinations | M01AB05 = diclofenac | N02AJ13 = tramadol and paracetamol | N02AC03 = piritramide |
| N02BG10 = cannabinoids | M01AB16 = aceclofenac | N02AJ06 = codeine and paracetamol | N02AC01 = dextromoramide |
|  | M01AB55 = diclofenac, combinations |  | N02AB03 = fentanyl |
|  | M01AC01 = piroxicam |  | N02AB02 = pethidine |
|  | M01AC06 = meloxicam |  | N02AA05 = oxycodone |
|  | M01AE01 = ibuprofen |  | N02AA04 =nicomorphine |
|  | M01AE02 = naproxen |  | N02AA03 = hydromorphone |
|  | M01AE03 = ketoprofen |  | N02AA01 = morphine |
|  | M01AE11 = tiaprofenic acid |  | N02AA55 = oxycodone and naloxone |
|  | M01AE52 = naproxen and esomeprazole |  |  |
|  | M01AH01 = celecoxib |  |  |
|  | M01AH05 = etoricoxib |  |  |
|  | M01AX01 = nabumetone |  |  |
|  | M01AX05 =glucosamine |  |  |

**Table S2:** subdivision of ICPC-2 codes by indication group included in the Poisson regression analyses

| **Group** | **ICPC-2 codes** |
| --- | --- |
| Musculoskeletal symptom or complaint | L07, L08, L09, L10, L11, L12, L13, L14, L15, L16, L17, L18, L19, L20, L27, L28, L29 |
| Musculoskeletal disease | L70, L82, L83, L84, L85, L87, L88, L94, L95, L97, L98, L99 |
| Back symptom or complaint | L02, L03 |
| Back syndrome with radiating pain | L86 |
| Osteoarthrosis | L89, L90, L91 |
| Trauma | A80, A81, A82, A84, A85, A86, A87, A88, A89, F75, F79, H77, H78, H79, L72, L73, L74, L75, L76, L77, L78, L79, L80, L81, L96, N79, N80, N81, R87, R88, S09, S10, S11, S12, S13, S14, S15, S16, S17, S18, S19, X82, Y80 |
| Malignancy | A79, B72, B73, B74, D74, D75, D76, D77, F74, L71, N74, N76, R84, R85, S77, T71, T73, U75, U76, U77, U79, X75, X76, X77, X81, Y77, Y78 |
| Headache | N01, N89, N90, N95 |
| Other disease | A70, A72, A75, A76, A77, A78, A90, A91, A92, A96, A97, A98, A99, B70, B75, B78, B80, B82, B83, B90, B99, D70, D71, D72, D73, D78, D81, D82, D83, D84, D85, D86, D87, D88, D89, D90, D91, D92, D93, D94, D95, D97, D98, D99, F70, F71, F72, F73, F82, F84, F91, F92, F93, F94, F95, F99, H70, H71, H72, H73, H74, H80, H81, H82, H84, H86, H99, K70, K71, K73, K74, K75, K76, K77, K78, K80, K81, K82, K83, K84, K85, K86, K87, K88, K89, K90, K91, K92, K93, K94, K95, K96, K99, N70, N71, N73, N75, N85, N86, N87, N88, N91, N92, N93, N94, N99, P70, P71, P73, P74, P75, P76, P77, P78, P79, P80, P81, P82, P85, P98, P99, R71, R72, R73, R74, R75, R76, R77, R78, R79, R80, R81, R82, R83, R86, R90, R95, R96, R97, R98, R99, S03, S70, S71, S72, S74, S76, S78, S79, S80, S81, S82, S84, S85, S86, S87, S88, S91, S92, S93, S94, S96, S97, S98, S99, T81, T82, T83, T85, T86, T80, T89, T90, T91, T92, T93, T99, U70, U71, U72, U78, U79, U85, U88, U95, U99, W70, W76, W78, W79, W80, W82, W83, W84, W85, W90, W92, W94, W95, W96, X72, X74, X78, X79, X80, X83, X84, X85, X86, X87, X88, X89, X90, X91, X92, X99, Y70, Y72, Y73, Y74, Y75, Y76, Y83, Y85, Y86, Y99 |
| Other symptom | A01, A03, A04, A05, A06, A08, A09, A10, A11, A13, A16,A18, A20, A21, A23, A27, A28, A29, B02, B28, D01, D02, D03, D04, D05, D06, D07, D08, D09, D10, D11, D12, D14, D15, D16, D19, D20, D21, D25, D26, D29, F01, F02, F04, F05, F13, F16, H01, H02, H03, H04, H05, H13, H15, K01, K02, K03, K04, K05, K07, K22, K25, K27, K28, N03, N04, N05, N06, N07, N08, N17, N18, N28, N29, P01, P02, P03, P04, P05, P06, P07, P09, P10, P15, P18, P19, P20, P22, P24, P27, P29, R01, R02, R03, R04, R05, R06, R07, R08, R09, R21, R23, R25, R27, R29, S01, S02, S04, S05, S06, S07, S08, S20, S21, S22, S23, S29, T03, T07, T08, T11, U01, U02, U04, U05, U06, U07, U08, U13, U14, U28, U29, W02, W10, W11, W12, W13, W14, W15, W17, W18, W19, W28, X01, X02, X03, X05, X06, X07, X08, X09, X10, X11, X12, X14, X15, X16, X17, X18, X19, X20, X21, X22, X23, X28, X29, Y01, Y02, Y03, Y04, Y05, Y06, Y07, Y13, Y16, Y25, Y29, Z01, Z03, Z05, Z10, Z12, Z13, Z15, Z16, Z18, Z23, Z25, Z29 |

**Table S3:** classification of ICPC-2 codes by indication group included in the multilevel regression analysis

| **Group** | **ICPC-2 codes** |
| --- | --- |
| Musculoskeletal disorders  (= osteoarthritis, rheumatoid arthritis and lower back pain) | L84, L86, L88, L89, L90, L91 |
| Psychological disorders  (= depressive disorder, anxiety disorder and neurasthenia/surmenage) | P74, P76, P78 |
| Malignancies | A79, B72, B73, B74, D74, D75, D76, D77, L71, N74, N76, R84, R85, S77, T71, T73, U75, U76, U77, U79, X75, X76, X77, X81, Y77, Y78 |

**Table S4:** classification of the variables included in the multilevel regression analysis

| **Variable** | **Definition** |
| --- | --- |
| Educational level | Low: primary school and lower general secondary education  Middle: higher general secondary education and intermediate vocational education  High: higher vocational education and university education |
| Alcohol consumption | None: 0 glasses a week  Moderate: women 1-7 glasses a week, men 1-14 glasses a week  Heavy: women >7 glasses a week, men >14 glasses a week |

**Table S5:** additional results of multilevel regression analysis

| **Fixed Coefficients** |  |  |  |  |  |  |  |  |
| --- | --- | --- | --- | --- | --- | --- | --- | --- |
| **Model Term** | Coefficient | Std. Error | Sig. | 95% Confidence Interval | | OR | 95% Confidence Interval for OR | |
|  |  |  |  | Lower | Upper |  | Lower | Upper |
| Intercept | -4,008 | 0,2727 | <,001 | -4,543 | -3,47 | 0,018 | 0,011 | 0,031 |
| Musculoskeletal disorder=1 | 0,876 | 0,1164 | <,001 | 0,648 | 1,104 | 2,401 | 1,911 | 3,016 |
| Musculoskeletal disorder=0 | ref |  |  |  |  |  |  |  |
| Psychological disorder=1 | 0,318 | 0,1148 | 0,006 | 0,093 | 0,543 | 1,374 | 1,097 | 1,721 |
| Psychological disorder=0 | ref |  |  |  |  |  |  |  |
| Malignancy=1 | 0,605 | 0,1473 | <,001 | 0,316 | 0,894 | 1,831 | 1,372 | 2,444 |
| Malignancy=0 | ref |  |  |  |  |  |  |  |
| Age | 0,009 | 0,0042 | 0,027 | 0,001 | 0,018 | 1,009 | 1,001 | 1,018 |
| SES |  |  | 0,01 |  |  |  |  |  |
| SES=2 (low) | 0,421 | 0,1406 | 0,003 | 0,145 | 0,696 | 1,523 | 1,156 | 2,006 |
| SES=1 (middle) | 0,282 | 0,1341 | 0,036 | 0,019 | 0,545 | 1,326 | 1,019 | 1,724 |
| SES=0 (high) | ref |  |  |  |  |  |  |  |
| Sex=1 (female) | 0,036 | 0,1168 | 0,76 | -0,193 | 0,265 | 1,036 | 0,824 | 1,303 |
| Sex=0 (male) | ref |  |  |  |  |  |  |  |
| Smoking status |  |  | <,001 |  |  |  |  |  |
| Smoking status=2 (current) | 0,796 | 0,1496 | <,001 | 0,503 | 1,09 | 2,218 | 1,654 | 2,974 |
| Smoking status=1 (former) | 0,42 | 0,1316 | 0,001 | 0,162 | 0,677 | 1,521 | 1,175 | 1,969 |
| Smoking status=0 (never) | ref |  |  |  |  |  |  |  |
| Marital state=1 (single) | 0,022 | 0,1485 | 0,882 | -0,269 | 0,313 | 1,022 | 0,764 | 1,368 |
| Marital state=0 (together) | ref |  |  |  |  |  |  |  |
| Alcohol consumption |  |  | <,001 |  |  |  |  |  |
| Alcohol consumption=2 (heavy) | -0,56 | 0,1633 | <,001 | -0,88 | -0,24 | 0,571 | 0,415 | 0,786 |
| Alcohol consumption=1 (moderate) | -0,708 | 0,1227 | <,001 | -0,949 | -0,47 | 0,493 | 0,387 | 0,627 |
| Alcohol consumption=0 (none) | ref |  |  |  |  |  |  |  |
| History of abuse=1 | 0,356 | 0,134 | 0,008 | 0,094 | 0,619 | 1,428 | 1,098 | 1,857 |
| History of abuse=0 | ref |  |  |  |  |  |  |  |
|  |  |  |  |  |  |  |  |  |
| **Random effect** |  |  |  |  |  |  |  |  |
| **Model term** |  |  |  |  |  |  |  |  |
| Intercept | 0,016 |  |  |  |  |  |  |  |

Model fit statistics:

Akaike Corrected: 41505,117

Bayesian 41511,953
